# Supplementary material for: A 2-Year Retrospective Case Series on Isolates of the Emerging Pathogen Actinotignum schaalii from a Canadian Tertiary Care Hospital
Source: Microorganisms. 2022 Aug 9;10(8):1608. doi: 10.3390/microorganisms10081608 (PMC9412865; doi:10.3390/microorganisms10081608)
Supplement: Supplementary file 1 [file microorganisms-10-01608-s001.zip › microorganisms-1824282-supplementary.pdf]

Supplementary Sheet: A 2-year Retrospective Case Series on isolates of the Emerging Pathogen Actinotignum schaalii from a Canadian tertiary care hospital

Authors:
Pramath Kakodkar, and Camille Hamula

Affiliation:
Department of Pathology and Laboratory Medicine, University of Saskatchewan, Canada

Corresponding Author:
Camille Hamula

Disclosures: The authors declare no conflicts of interest

Financial Support: Not applicable

Table S1: Summary of Comorbidities in our case series.

	**Comorbidity (Procedure)**	***n***
Endocrine (25)	Hyperthyroidism	1
	Hypothyroidism	6
	Type 2 Diabetes Mellitus	9
	Systemic Lupus erythematosus	1
	Obesity/obstructive sleep apnea	8
Psychiatric (13)	Anxiety	2
	Depression	2
	Dementia/Delirium	9
Gastroenterology (24)	Pancreatitis	1
	Gastroesophageal reflux disease	9
	Cirrhosis	1
	Cholecystectomy	2
	Hernia	2
	Hemorrhoids (hemorrhoidectomy)	2
	diverticulitis (colostomy)	2
	Inflammatory bowel disease (subtotal colectomy and end-ileostomy)	2
	Appendectomy	3
Renal (7)	Renal Failure	5
	Gout	2
Woman's' health (5)	Breast cancer	1
	Gynecology procedures	4
Others (11)	Arthritis	6
	Chronic obstructive pulmonary disease	4
	Foot cellulitis	1
Hematology (17)	Deep vein thrombosis	1
	Provoked pulmonary embolism	2
	Anemia	3
	Superficial arm thrombophlebitis	2
	Peripheral vascular disease	1
Urology (12)	Urolithiasis	1
	Urinary tract infection	3
	Bladder pathologies	3
	BPH	3
	Prostate ca	2
Orthopedic (3)	Knee replacement	1
	Hip fracture	2
Cardiology (29)	Heart failure with reduced ejection fraction	4
	hypertension	16
	Aortic Dissection	1
	Abdominal aortic aneurysm	1
	Atrial fibrillation	5
	Coronary artery disease	2
Neurology (14)	Cerebral vascular accident/ transient ischemic attack	4

	Movement disorders	7
	Cerebral palsy	1
	Spina bifida	2

Table S2: Literature review summary of *A. schaalii* urological infections.

Age	Diagnosis	Mono/polymicrobial; organism	Treatment	References
**Pyelonephritis (12),** Mean = 51±24.8, M:F (8:4), Blood (5), Urine (6), Aspirate (1)	Pyelonephritis (12)	Mono (8), Poly (4); *Bacteroides fragilis, Escherichia coli, Candida albicans, , Peptoniphilus asaccharolyticus, Actinomyces odontolyticus.*	Ampicillin (3), Amoxiclav (2), ciprofloxacin (2), PIP-TZM (2), CTX, gentamicin, Amoxicillin, Vancomycin, TMP-SMX, Nephrostomy (2), JJ stent (2), Pyeloplasty, Surgical drainage, Nephrectomy	[1], [2], [3], [4], [5], [6], [7],[8] *
**UTI (65),** Mean = 73±2.9, M:F (39:26), Urine (45), Blood (7), Urine and blood (6), Aspirate (), Semen (2)	UTI unspecified (17)	Mono (9), Poly (8); *Klebsiella pneumonia, Corynebacterium, Aerococcus sanguinicola,* Urethral flora, Gram+ve mixed flora (3), CoNS	PIP-TZM (3), erythromycin, Ciprofloxacin (5), Metronidazole (2), Clindamycin, Fosfomycin, cefuroxime, Amoxiclav (2), Cefotaxime (4), Cefadroxil, Levofloxacin, CTX, Cephalexin, Ceftazidime, gentamicin,	[9], [10], [11], [12],
	Acute urinary retention (7)	Mono (3), Poly (4); *Aerococcus urinae* (2), *Escherichia coli, Enterobacter cloacae,* Urethral flora	Ciprofloxacin, Cefotaxime (3), amoxicillin, suprapubic catheterization.	[9], [13]
	Prostatitis (9)	Mono (4), Poly (5); *Escherichia coli* (2), *Actinomyces neuii, Pseudomonas aeruginosa, Candida glabrata*	Cefixime, Ceftriaxone, amoxiclav (3), Doxycycline, amoxicillin (4), Ciprofloxacin (3), Azithromycin, Penicillin G, cefpodoxime	[4], [14], [12], [15]
	Cystitis (17)	Mono (9), Poly (8); *Aerococcus urinae, Staphylococcus aureus, Escherichia coli* (2), *Citrobacter koseri, Klebsiella pneumoniae, Staphylococcus*	Nitrofurantoin, ampicillin, Cefuroxime, Amoxicillin (3), Amdinocillin, TMP-SMX (2), Amoxiclav (3), Ciprofloxacin (2), Norfloxacin, Pivampicillin	[13], [2], [16], [17], [18], [4]

		epidermidis, Enterococcus faecalis	(2), mecillinam, CTX, catheter change	
	Dementia (6)	Mono (5), Poly (1); *Aerococcus urinae*	Metronidazole (2), CTX, Amoxicillin, Cefuroxime	[13], *
	Neurogenic bladder (3)	Mono (3)	Amoxiclav (2), TMP-SMX, Amoxicillin	[19], *
	Urethritis (2)	Mono (2)	Amoxicillin (2)	[4]
	Bladder Necrosis (non-muscle-invasive bladder cancer) (2)	Mono (2)	Amoxicillin (2)	[4], [20]
	Bladder Prolapse (2)	Mono (1), Poly (1); *Aerococcus urinae*	Cystoscopy (2), Amoxicillin, Nitrofurantoin, Ciprofloxacin	*

*Patients included from our case series.

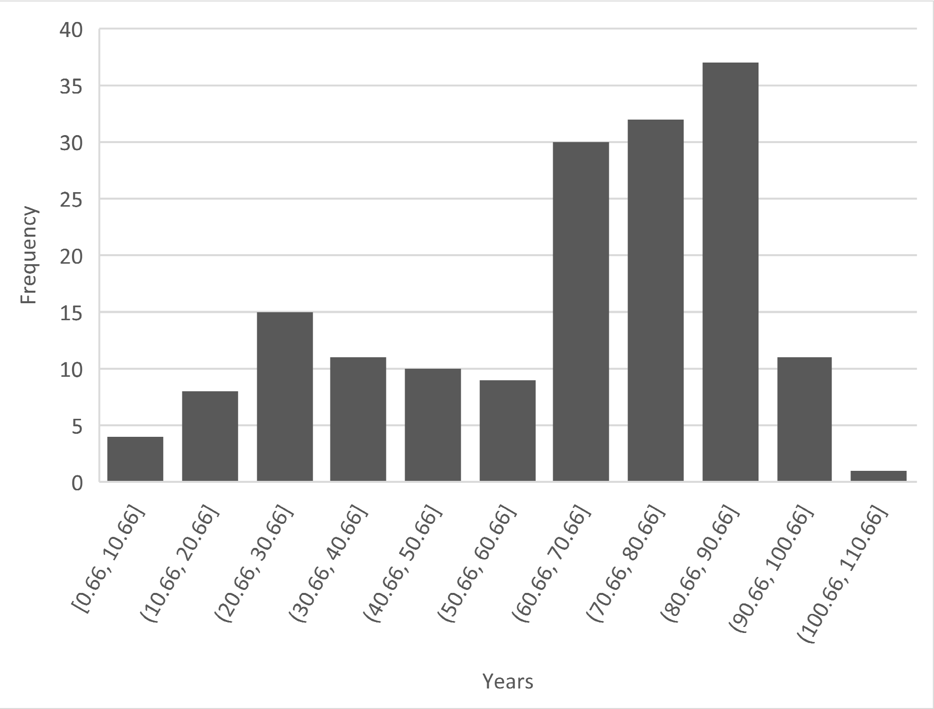

Supplementary Figure S1: Population distribution of patient cohort from the Literature review (*n*=134)

References

1. Lawson, P.A., et al., *Characterization of some Actinomyces-like isolates from human clinical specimens: reclassification of Actinomyces suis (Soltys and Spratling) as Actinobaculum suis comb. nov. and description of Actinobaculum schaalii sp. nov.* Int J Syst Bacteriol, 1997. **47**(3): p. 899-903.
2. Reinhard, M., et al., *Ten cases of Actinobaculum schaalii infection: clinical relevance, bacterial identification, and antibiotic susceptibility.* J Clin Microbiol, 2005. **43**(10): p. 5305-8.
3. Barberis, C., et al., *Actinobaculum schaalii causing urinary tract infections: report of four cases from Argentina.* J Infect Dev Ctries, 2014. **8**(2): p. 240-4.
4. Lotte, L., et al., *Infections related to Actinotignum schaalii (formerly Actinobaculum schaalii): a 3-year prospective observational study on 50 cases.* Clin Microbiol Infect, 2016. **22**(4): p. 388-390.
5. Gupta, A., P. Gupta, and A. Khaira, *Actinobaculum schaalii pyelonephritis in a kidney allograft recipient.* Iran J Kidney Dis, 2012. **6**(5): p. 386-8.
6. Pajkrt, D., et al., *Pyelonephritis caused by Actinobaculum schaalii in a child with pyeloureteral junction obstruction.* Eur J Clin Microbiol Infect Dis, 2003. **22**(7): p. 438-40.
7. Vallet, A., et al., *Recurrent obstructive acute pyelonephritis: A rare form of Actinotignum (Actinobaculum) schaalii infection in a HIV-1 infected patient.* Anaerobe, 2017. **43**: p. 75-77.
8. Kitano, H., et al., *Case Report: Emphysematous Pyelonephritis With a Congenital Giant Ureterocele.* Front Pediatr, 2021. **9**: p. 775468.
9. Sandlund, J., et al., *Bacteraemia caused by Actinobaculum schaalii: An overlooked pathogen?* Scand J Infect Dis, 2014. **46**(8): p. 605-8.
10. Larios, O.E., et al., *First report of Actinobaculum schaalii urinary tract infection in North America.* Diagn Microbiol Infect Dis, 2010. **67**(3): p. 282-5.
11. Vasquez, M.A., et al., *Actinobaculum schaalii recurrent urinary infection in a centenarian patient.* Geriatr Gerontol Int, 2013. **13**(3): p. 807-8.
12. Gomez, E., et al., *Actinobaculum bacteremia: a report of 12 cases.* Journal of clinical microbiology, 2011. **49**(12): p. 4311-4313.
13. Bank, S., et al., *A validation of the Danish microbiology database (MiBa) and incidence rate of Actinotignum schaalii (Actinobaculum schaalii) bacteraemia in Denmark.* Clin Microbiol Infect, 2015. **21**(12): p. 1097.e1-4.
14. Siller Ruiz, M., et al., *Unusual clinical presentations of Actinotignum (Actinobaculum) schaalii infection.* Enferm Infecc Microbiol Clin, 2017. **35**(3): p. 197-198.
15. van Aarle, S., N.L.A. Arents, and K. de Laet, *Actinobaculum schaalii causing epididymitis in an elderly patient.* J Med Microbiol, 2013. **62**(Pt 7): p. 1092-1093.
16. Haller, P., et al., *Vertebral osteomyelitis caused by Actinobaculum schaalii: a difficult-to-diagnose and potentially invasive uropathogen.* Eur J Clin Microbiol Infect Dis, 2007. **26**(9): p. 667-70.
17. Sturm, P.D., et al., *Urosepsis with Actinobaculum schaalii and Aerococcus urinae.* J Clin Microbiol, 2006. **44**(2): p. 652-4.
18. Andersen, L.B., et al., *Actinobaculum schaalii, a cause of urinary tract infections in children?* Acta Paediatr, 2012. **101**(5): p. e232-4.
19. Zimmermann, P., et al., *Actinobaculum schaalii an emerging pediatric pathogen?* BMC Infect Dis, 2012. **12**: p. 201.
20. Lotte, R., et al., *A rare case of histopathological bladder necrosis associated with Actinobaculum schaalii: the incremental value of an accurate microbiological diagnosis using 16S rDNA sequencing.* Anaerobe, 2014. **26**: p. 46-8.
